# Tactile feedback improves auditory spatial localization

**DOI:** 10.3389/fpsyg.2014.01121

**Published:** 2014-10-20

**Authors:** Monica Gori, Tiziana Vercillo, Giulio Sandini, David Burr

**Affiliations:** ^1^Robotics Brain and Cognitive Sciences Department, Istituto Italiano di TecnologiaGenoa, Italy; ^2^Department of Neuroscience, Psychology, Pharmacology and Child Health, University of FlorenceFlorence, Italy

**Keywords:** recalibration, auditory localization, spatial perception, tactile feedback

## Abstract

Our recent studies suggest that congenitally blind adults have severely impaired thresholds in an auditory spatial bisection task, pointing to the importance of vision in constructing complex auditory spatial maps (Gori et al., 2014). To explore strategies that may improve the auditory spatial sense in visually impaired people, we investigated the impact of tactile feedback on spatial auditory localization in 48 blindfolded sighted subjects. We measured auditory spatial bisection thresholds before and after training, either with tactile feedback, verbal feedback, or no feedback. Audio thresholds were first measured with a spatial bisection task: subjects judged whether the second sound of a three sound sequence was spatially closer to the first or the third sound. The tactile feedback group underwent two audio-tactile feedback sessions of 100 trials, where each auditory trial was followed by the same spatial sequence played on the subject’s forearm; auditory spatial bisection thresholds were evaluated after each session. In the verbal feedback condition, the positions of the sounds were verbally reported to the subject after each feedback trial. The no feedback group did the same sequence of trials, with no feedback. Performance improved significantly only after audio-tactile feedback. The results suggest that direct tactile feedback interacts with the auditory spatial localization system, possibly by a process of cross-sensory recalibration. Control tests with the subject rotated suggested that this effect occurs only when the tactile and acoustic sequences are spatially congruent. Our results suggest that the tactile system can be used to recalibrate the auditory sense of space. These results encourage the possibility of designing rehabilitation programs to help blind persons establish a robust auditory sense of space, through training with the tactile modality.

## INTRODUCTION

Many studies show that vision is fundamental for space perception. For example, when vision and sound are in conflict, vision usually dominates, causing the so-called “ventriloquist effect” (Warren et al., 1981; Mateeff et al., 1985). Vision not only dominates sound in spatial location (under most conditions: Alais and Burr, 2004), it can also affect audition over longer periods. For example, owls reared with distorting prisms show systematic and persistent biases in auditory localization (Knudsen and Knudsen, 1985), that persist after removal of the lenses. Comparable (but transitory) effects have also been demonstrated in humans, after relatively short periods of adaptation to systematically non-aligned auditory and visual stimuli (Recanzone, 1998; Zwiers et al., 2003).

It has been shown that in absence of vision it is possible to develop some auditory spatial skills. For example studies show that blind individuals have enhanced auditory skills for static sound localization or for discriminating the relation between two sounds in the horizontal axis (e.g., Lessard et al., 1998; King and Parsons, 1999; Roder et al., 1999; Gougoux et al., 2004; Doucet et al., 2005; Lewald, 2007). This enhancement can reflect changes in the auditory pathway (e.g., Korte and Rauschecker, 1993; Elbert et al., 2002) or the recruitment of visual cortex (e.g., Weeks et al., 2000; Gougoux et al., 2005; Poirier et al., 2005; Renier and De Volder, 2005; Striem-Amit and Amedi, 2014).

On the other hand, concerning the understanding of the relationship between three sounds during a space bisection task, it has been recently shown that congenitally blind humans show specific deficits (Gori et al., 2014). This result is in agreement with the fact that vision plays an important role in auditory space calibration. It is also in agreement with neurophysiological studies showing that vision guides the maturation of auditory spatial response properties of neurons of superior colliculus (e.g., King et al., 1988; Knudsen and Brainard, 1991; King and Carlile, 1993; Wallace and Stein, 2007).

These studies raise the question of whether it may be possible to develop strategies to help reconstruct the auditory sense of space in the congenitally blind, via a different sensory modality, such as touch or audition. Evidence from blind echolocators supports this idea. Echolocation is the extraordinary ability to represent the external environment by using reflected sound waves from self-generated auditory pulses. Some blind humans who echolocate by making mouth clicks and listening to the echoes demonstrate excellent spatial acuity (Thaler et al., 2011, 2014; Teng et al., 2012). Unfortunately this is a rare ability that only few people naturally develop. Technology has also tried to move in this direction proposing different kinds of sensory substitution devices (SSDs) for visually impaired individuals. The empirical and experimental results deriving from the use of these devices shows that it is possible, to some extent, to signal visual information to the blind using haptic or auditory modalities (see Bach-y-Rita and Kercel, 2003 for a review). Interestingly, it has been shown that in some cases the substitution devices produce direct effect on cortical plasticity (e.g., Striem-Amit and Amedi, 2014). Interestingly, the most popular sensory displays substitute vision with tactile signals, applied to various surface areas to provide electro- and vibrotactile-vision sensory substitution (see Bach-y-Rita and Kercel, 2003 for a review). However, the neural mechanisms underlying these sensory substitutions are poorly understood.

That the tactile signal can successfully substitute vision in SSD suggests that this modality could be used to recalibrate the auditory sense of space in the absence of vision. To test this hypothesis we measured auditory spatial perception in 48 blindfolded sighted subjects, before and after audio-tactile feedback. Subjects’ performance improved significantly after tactile feedback, but only when the sound and the tactile stimulations are spatially coherent. This result supports the idea that direct tactile feedback can interact with auditory spatial representation, possibly via a recalibration mechanism.

## MATERIALS AND METHODS

**FIGURE 1:**
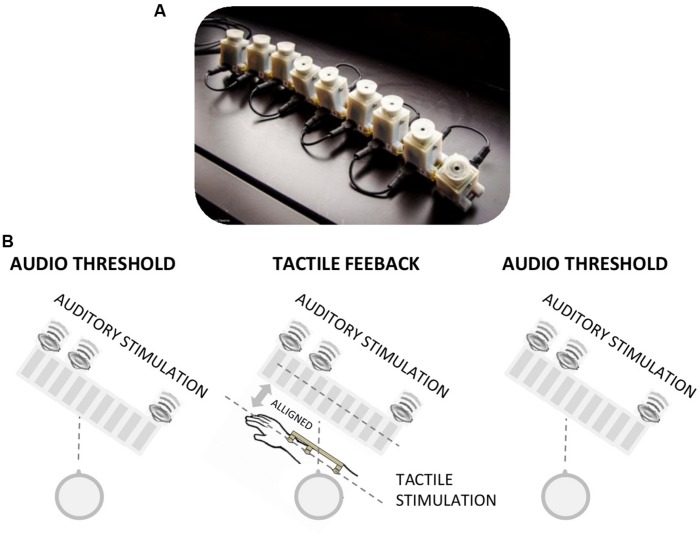
**(A)** Image of the vibrotactile devices used for the tactile feedback. The device comprises a series of vibrotactile units (on the underside), each of which can be driven individually. **(B)** Image representing the tactile feedback condition. The audio spatial threshold was first measured by the bisection technique. They were then given a first session of audio-tactile feedback, the spatial audio threshold was measured again, a second session of audio-tactile feedback and the spatial audio threshold was then repeated.

We tested 48 sighted subjects (age: 24.8 ± 0.6 years). Participants were blindfolded before entering the room, so they had no notion of the room or speaker layout. They were sat at the center of a bank of nine speakers, spanning ±17.5° of visual angle, aligned with the fifth speaker (at the center of the array), 90 cm away. In order to decrease auditory precision (to allow for more improvement), we positioned the array obliquely with respect to the subjects (see **Figure 1**). Subjects were assigned at random to one of five groups: tactile feedback (*n* = 11); verbal feedback (*n* = 11); no feedback (*n* = 14); rotated (*n* = 5); rotated–reversed (*n* = 7).

**FIGURE 2:**
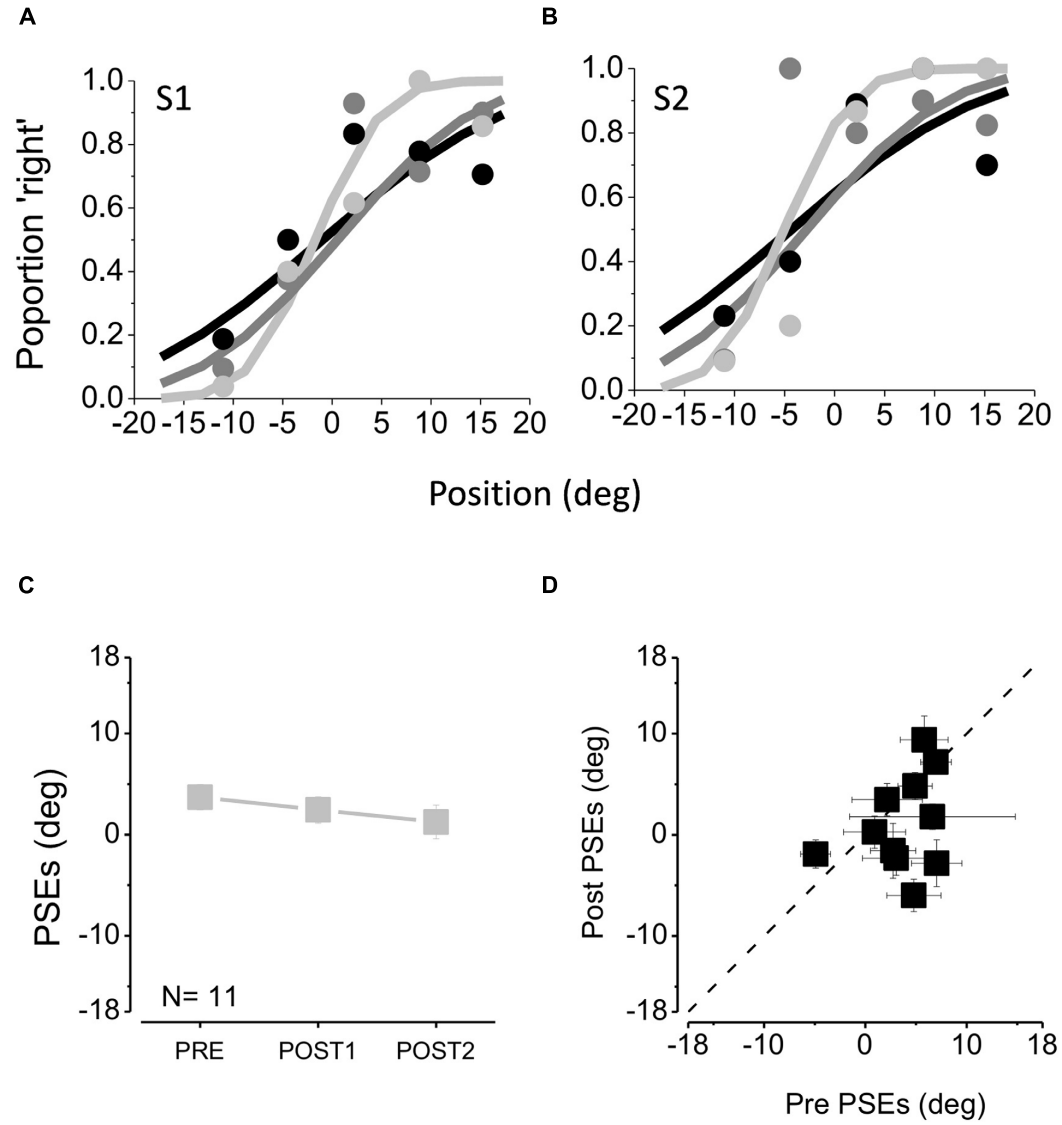
**(A,B)** Psychometric functions of two example subjects, plotting proportion of trials where the middle sound is judged closer to the right-hand one, as a function of the position of this sound. The data were fit with cumulative Gaussian error functions, whose mean (50% point) gives the “point of subjective equality” (PSE) and steepness (SD) the estimate of threshold. Black data and curve are taken before feedback, the dark gray symbols after the first tactile feedback session and the light gray after the second tactile feedback session. Steeper psychometric functions suggest higher auditory precision. **(C)** Average PSEs measured before any feedback (PRE), after the first feedback session (POST1) and after the second feedback session (POST2). **(D)** PSEs after the second feedback session (POST2) against the initial PSEs (PRE).

Auditory spatial precision was measured by a bisection technique. Three brief sounds (500 Hz, 75 ms duration, 60 dB SPL at the subject) were presented successively at 500 ms intervals in three different positions. The first sound was always positioned at –17.5°, the third at +17.5°, and the second at an intermediate position determined by the QUEST adaptive algorithm (Watson and Pelli, 1983), which estimates point of subjective equality (PSE) after each response, and places the next trial near that estimate. To ensure that a wide range of positions was sampled, that estimate was jittered by a random amount, drawn from a Gaussian distribution of space constant 17.5°, and the nearest speaker to that estimate chosen. Subjects reported verbally whether the second sound was closer to the left (speaker 1 at –17.5°) or right sound (speaker 9 at +17.5°). To ensure that a wide range of positions was sampled, that estimate was jittered by a random amount, drawn from a Gaussian distribution of space constant 17.5°, and the nearest speaker to that estimate chosen. Each subject performed 100 trials for each measure of threshold. The proportion of rightward responses was plotted as a function of the speaker position, and the data fit with a cumulative Gaussian function (see **Figure 2**) by means of the maximum likelihood method to estimate both PSE (given by the mean) and threshold (SD). The space constant (σ) of the fit was taken as the estimate of threshold indicating precision for the bisection task.

The paradigm comprised five phases: initial measurement of bisection threshold; first feedback phase; second measurement of bisection threshold; second feedback phase; final measurement of bisection threshold. During the feedback phases, subjects were presented a sequence of three sounds like the testing sequence. However, during this phase the subject did not respond, but merely paid attention to the sound sequence in order to associate this with the following feedback. To monitor attention, we randomly presented 10 higher tones within the 100 trials which subjects had to detect.

Feedback was either *tactile* or *verbal*, with 100 feedback trials in each feedback phase. In the tactile feedback condition, each of the nine speakers was associated with one of nine aligned vibrotactile stimulators (**Figure 1A**) positioned in the forearm of the subjects, and aligned spatially with the speakers (i.e., the arm of the subject was positioned parallel with respect to the speaker array with the middle tactile stimulus positioned in front of the middle speaker: **Figure 1B**). Each auditory three sound sequence was followed by the same tactile spatial sequence on the subjects’ arm, after 200 ms delay. As the subject was positioned facing the speaker array, the audio and tactile spatial positions were coherent, in the same line of sight. In the verbal feedback condition, the positions of the sounds were verbally reported to the subjects by the experimenter after each presentation. The experimenter verbally reported the sequence of the speakers that produced the sound (e.g., “number 1, 3, 9” as represented in **Figure 1B**). Before testing, also in this experimental condition, the subject was informed that the first sound was always produced by the first speaker (number 1) and the last sound by the last speaker (number 9). In the no feedback condition, only the three testing phases were performed, with no feedback sessions.

Two further conditions were run to examine the importance of spatial coherence. One we refer to as the *rotated* condition, where tactile feedback was provided, but with the subjects rotated 180° so the speakers were positioned behind the subject. In this condition the position of the sounds and of the tactile stimulations were reversed in space. Another condition we refer to as *rotated–reversed*, where the subjects were rotated 180° with respect to the speakers, but the order of the tactile stimulators was reversed so they were aligned with the order of the speakers.

At the beginning of the testing session, all subjects (blindfolded before entering in the room) were described the setup, the stimuli presentation and the number of speakers. In the tactile, verbal, and no feedback conditions the arm of the participants was in the same position of the tactile feedback condition (see **Figure 1B**), even if the tactile device was not positioned on the arm. In the rotated and rotated–reversed conditions, the arm of the subject was reflected with respect to the speaker array. To check that the tactile precision was similar between groups in the tactile, rotated, and rotated–reversed group we also measured tactile bisection threshold at the end of the entire sessions. The task was identical to the auditory one with the only exception that the stimulation was provided by the vibrotactile devices positioned in the subject arm (**Figure 1A**). All participants gave informed consent prior to testing. The study was approved by the ethics committee of the local health service (*Comitato Etico, ASL3, Genova*).

## RESULTS

**Figures 2A,B** show the psychometric functions for two example subjects (S1 and S2) at the beginning of the session (black line), after the first tactile feedback block (dark gray line) and after the second tactile feedback block (light gray line). The curves plot the proportion of trials where the middle sound is perceived closer to the third sound. The data have been fit with cumulative Gaussian functions, whose mean (50% point) gives the “point of subjective equality,” or PSE, the perceptual midpoint of the speakers.

**Figure 2C** plots the average points of subjective equality as a function of tactile feedback session, showing that tactile feedback had very little effect on PSE, causing only a slight tendency to reduce the small positive bias. **Figure 2D** plots the individual PSEs after two session of feedback against the pre-feedback values. The points cluster around the equality line, with no significant difference with respect to the pre-training session after the first (one tailed paired *t*-test, *t*_10_ = 0.90, *p* = 0.19) and between the first and the second training sessions (one tailed paired *t*-test, *t*_10_ = 1.01, *p* = 0.16).

**FIGURE 3:**
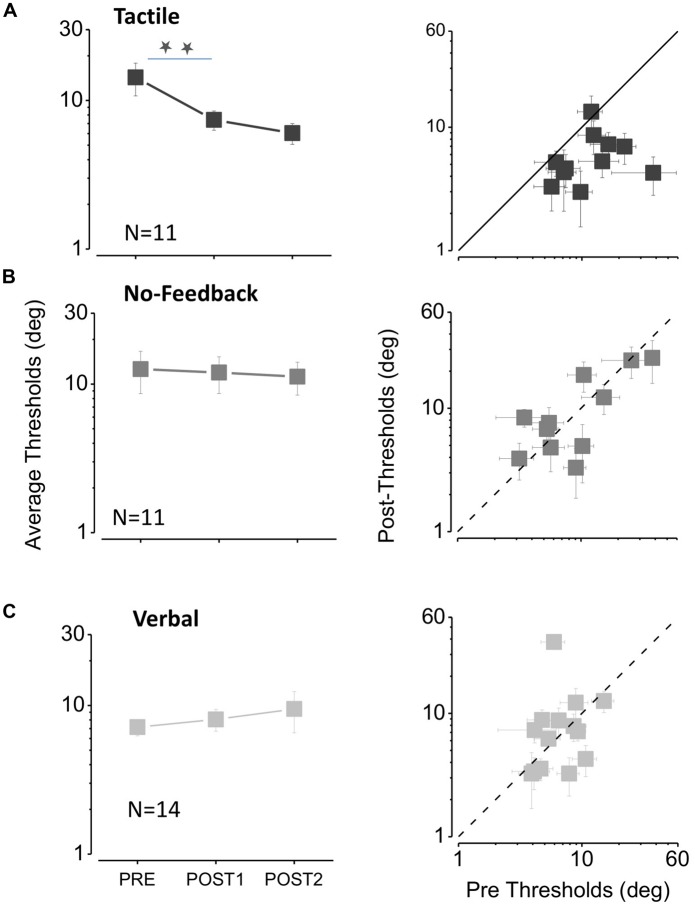
**The effect of feedback on auditory bisection thresholds. (A)** Tactile feedback. The three data points in the left-hand graph plot average thresholds measured before the feedback sessions (PRE), after the first feedback session (POST1) and after the second session (POST2). The stars indicate a significant difference level of *p* < 0.05 (one tailed *t*-test, *p* = 0.02 after the first feedback; one tailed *t*-test, *p* = 0.01 after the second feedback). The plot at right shows the thresholds for all subjects, plotting the thresholds after the second feedback (POST2) against the initial thresholds (PRE). All points fall below the equality line, showing that all subjects improved after feedback sessions. **(B)** Same as **(A)** for the no feedback condition. **(C)**. Same as **(A)** for the verbal feedback condition.

Inspection of the curves shows that the psychometric functions become steeper after the tactile feedback sessions, suggesting that precision increases after auditory-tactile spatial association. We take the steepness of the curve (given by the SD) as the estimate of thresholds. **Figure 3** plots thresholds for the tactile, no feedback and verbal conditions. The graphs at left show average results, and those at right individual thresholds. Tactile feedback (**Figure 3A** on the left) caused a clear and significant improvement with feedback [repeated measures multi-comparison one way analysis of variance (ANOVA) *F*(2,30) = 5.18, *p* = 0.011]. Thresholds decreased from 14.3 ± 3.5° before feedback to 7.4 ± 1.1° after the first session (two tailed paired *t*-test, *t*_10_ = 2.3, *p* = 0.04), and to 6.0 ± 1.0° after the second session (two tailed paired *t*-test, *t*_10_ = 2.63, *p* = 0.02). **Figure 3A** on the right shows that thresholds improved for almost all subjects, with all except one data point falling below the equality line.

Improvement was specific to the tactile feedback condition. It did not occur spontaneously (**Figure 3B**), nor with verbal feedback (**Figure 3C**). Neither showed significant improvement (repeated measures multi-comparison one way ANOVA *F*(2,30) = 0.03, *p* = 0.97 for no feedback and ANOVA *F*(2,39) = 0.34, *p* = 0.71 for verbal feedback). In both cases the individual data points are scattered around the equality line, with no tendency to fall below it.

**FIGURE 4:**
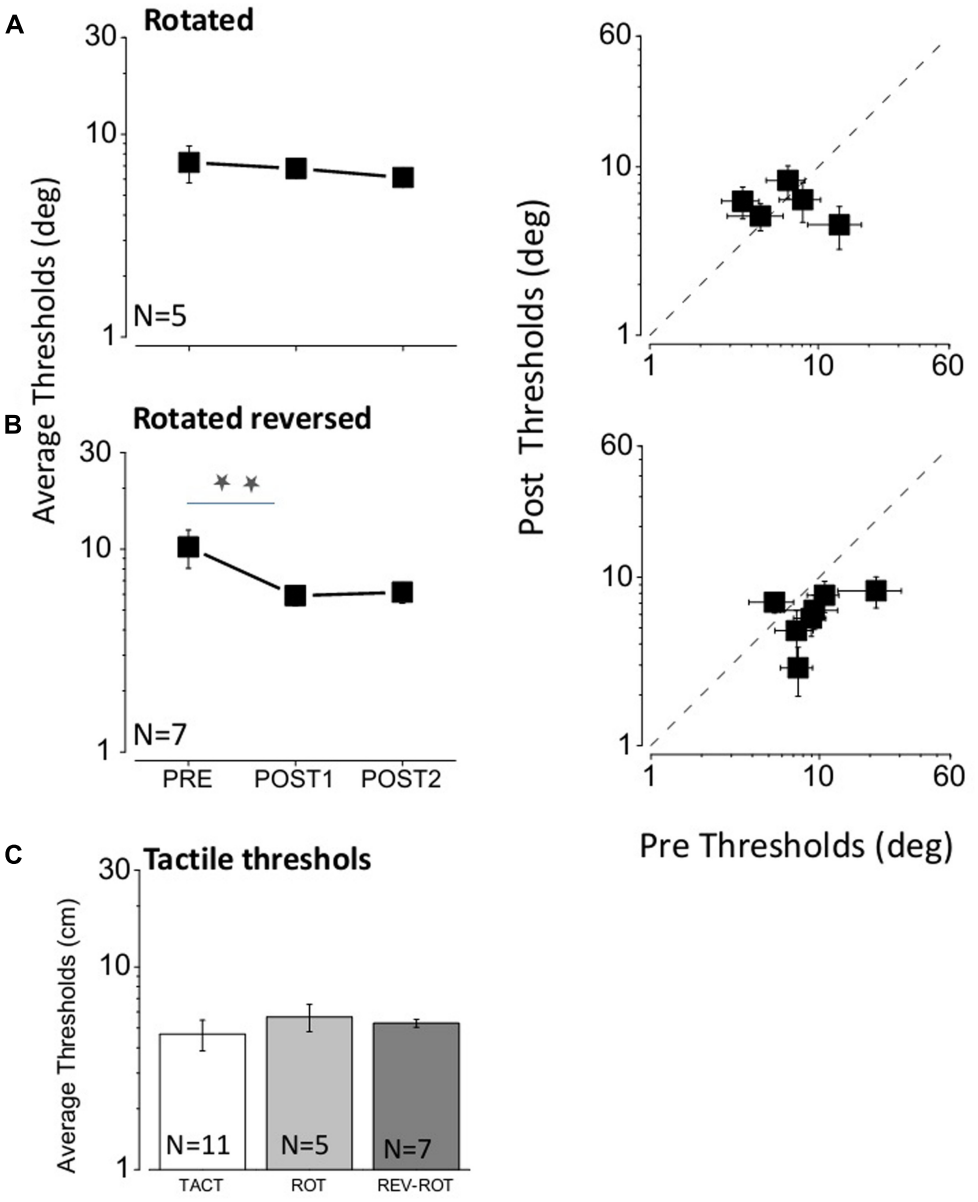
**(A)** Results for the rotated feedback condition. The three data points in the left-hand graph represent the average results for the three bisection thresholds measured: the first one before any feedback (PRE), the second after the first feedback (POST1) and the third after the second feedback (POST2). The plot at right shows the thresholds for all subjects, plotting the thresholds after the second feedback (POST2) against the initial thresholds (PRE). **(B)** Same as **(A)** for the rotated–reversed feedback condition. Two stars represent a significant difference level of <0.05 (one tailed *t*-test, *p* = 0.04 after the first feedback; one tailed *t*-test, *p* = 0.03 after the second feedback). Also in this case all points, with the exception of one, fall below the equality line, showing that all subjects improved after feedback session. **(C)** Average tactile thresholds for the group of subjects with tactile, rotated and reverse–rotated feedback.

We then examined the importance of spatial coherence for the tactile feedback: in one condition (*rotated*) we rotated the subject, but left the ordering of the speakers as before (so it was reversed with respect to the direction of sound); in the other we rotated the subject but reversed the order of tactile stimulators so they corresponded with the direction of sound (*rotated–reversed*). The results are shown in **Figure 4**, in the same format as **Figure 3**. In the *rotated* condition (**Figure 4A**), the feedback has no effect [repeated measures multi-comparison one way ANOVA *F*(2,14) = 0.24, *p* = 0.79] after the first (one tailed paired *t*-test, *t*_4_ = 0.34, *p* = 0.37) and nor after the second training (one tailed paired *t*-test, *t*_4_ = 0.53, *p* = 0.3). However, in the *rotated–reversed* condition (**Figure 4B**), there was a significant improvement (repeated measures multi-comparison one way ANOVA *F*(2,20) = 3.38, *p* = 0.056) after the first (one tailed paired *t*-test, *t*_6_ = 2.17, *p* = 0.036) and after the second training (one tailed paired *t*-test, *t*_6_ = 2.3, *p* = 0.03), although less than when subjects faced the speakers (an average factor pre/post of 1.7 was obtained for the rotated and reversed condition while an average factor pre/post of 2.4 was obtained for the tactile condition). Also in this condition no change was observed for PSEs [repeated measures multi-comparison one way ANOVA *F*(2,20) = 0.87, *p* = 0.437] after the two feedback sessions (one tailed paired *t*-test, *t*_6_ = 1.23, *p* = 0.13 for the first feedback session and one tailed paired *t*-test, *t*_6_ = 1.5, *p* = 0.09 for the second feedback session). In order to check for tactile precision differences between groups we also measured the bisection task in the tactile modality (**Figure 4C**). No difference was found between subjects [repeated measures multi-comparison one way ANOVA *F*(2,20) = 0.49, *p* = 0.62]. Overall these results suggest that spatial correspondence is essential for the tactile feedback to improve auditory spatial localization.

## DISCUSSION

Audition, vision, and touch encode spatial information in different ways, from different sensor platforms. How our brain interprets these spatial cues within a common framework is still unclear. One possible way to keep the different sensory signals in alignment may be to use one system to *calibrate* the others. It has been suggested that best system to calibrate the others should be the more robust one, with the more accurate signals (Gori et al., 2008; Burr and Gori, 2011). For the perception of space, much evidence suggests that the visual system calibrates the others (Knudsen and Knudsen, 1985; Knudsen and Brainard, 1991; King and Carlile, 1993; Recanzone, 1998; Zwiers et al., 2001, 2003; Gori et al., 2008, 2010, 2012a,b; Burr and Gori, 2011). Vision seems to impact directly on the development of spatial aspects of other sensory systems (Gori et al., 2014).

In this study we showed that tactile feedback can improve auditory spatial discrimination in blindfolded sighted individuals. Auditory spatial precision improved by a factor of 2 after a brief feedback session. A second feedback session produced significant further improvement to a factor of 2.4 after the second training. We suggest that the improvement may reflect calibration (Burr and Gori, 2011) of the auditory sense of space, by tactile signals.

To control that the improvement in precision did not merely result from experience at the task, or from generic feedback, we incorporated two controls: one group received no feedback, and the other verbal feedback. Neither of these groups improved in performance, pointing to the importance of the feedback being of a sensory nature, rather than just being informative. Furthermore, to be effective, the feedback had to be spatially coherent with the sound source. When subjects were rotated by 180° so the spatial order was inverted on the arm, the feedback was ineffectual. However, when the order of the stimulators on the arm was reversed (restoring spatial coherence), the feedback became effective again. Clearly, to be effective, the feedback needs to be sensory. The lack of improvement observed in the verbal condition suggests that sensory, rather than cognitive mechanisms are involved. More interestingly, the feedback needs to have a spatial correspondence with the sound source to promote calibration between the tactile and the auditory system.

Cross-comparison between senses is clearly an effective strategy to establish and to maintain calibration, as each sense has access to different sources of information, differently affected by noise and distortions. Interestingly, our results suggest that in absence of one calibration modality, such as vision, for space, another modality can substitute it, and serve to calibrate the less robust modality (in this case audition).

The number of people with visual impairment worldwide in 2002 was over 161 million, of whom about 37 million were legally blind (Resnikoff et al., 2002). To date many technological solutions have been developed for visual disability. Several devices (both academic and commercial) available today are based on visual tactile substitution (e.g., Kaczmarek and Bach-Y-Rita, 1995; Bach-y-Rita et al., 1998; Kajimoto et al., 2003); and other on auditory-visual substitution (e.g., Meijer, 1992; Capelle et al., 1998). Our results suggest that stimulation of tactile modality can provide important information to recalibrate the sense of space in the absence of vision, and support the idea that both rehabilitation programs and SSD could provide tactile stimulation to substitute for vision. Further studies will be necessary to understand how these signals should be provided to produce the better effects in the everyday life of the visually impaired population.

## Conflict of Interest Statement

The authors declare that the research was conducted in the absence of any commercial or financial relationships that could be construed as a potential conflict of interest.
